# Psychosomatic symptoms questionnaire (PSQ-39): a psychometric study among general population of Iranian adults

**DOI:** 10.1186/s12888-021-03278-z

**Published:** 2021-05-25

**Authors:** Zahra Heidari, Awat Feizi, Sara Rezaei, Hamidreza Roohafza, Peyman Adibi

**Affiliations:** 1 Department of Biostatistics and Epidemiology, School of Health, Isfahan University of Medical Sciences, P.O. Box 319, Hezar-Jerib Ave, Isfahan, 81746- 73461 Iran; 2 Cardiac Rehabilitation Research Center, Cardiovascular Research Institute, Isfahan University of Medical Sciences, Isfahan, Iran; 3 Student Research Committee, School of Health, Isfahan University of Medical Sciences, Isfahan, Iran; 4 Gastroenterology and Hepatology Research Center, Isfahan University of Medical Sciences, Isfahan, Iran; 5 Department of Internal Medicine, School of Medicine, Isfahan University of Medical Sciences, Isfahan, Iran

**Keywords:** Psychosomatic symptoms, Bodily distress syndrome, Medically unexplained symptoms, Validity, Reliability, Psychometrics

## Abstract

**Background:**

Psychosomatic symptoms, characterized by physical-bodily complaints not fully explained by organic reasons, are highly prevalent. The present study aimed to culturally adapt and evaluate the psychometric properties of Psychosomatic Symptoms Questionnaire 39-item version (PSQ-39) among Iranian general adult population.

**Methods:**

This study included 996 Persian-speaking people, living in Isfahan, Iran. The translation of the PSQ-39 was performed using the forward-backward method. Test-retest reliability was evaluated through Intraclass correlation (ICC) coefficient and internal consistency by using Cronbach’s α. Construct validity was investigated by using both exploratory (EFA) and confirmatory (CFA) factor analysis. Short Form Health Survey (SF-36) was used to assess divergent validity. Known-group validity was also assessed.

**Results:**

The Persian version of the PSQ-39 showed excellent test-retest reliability in all domains (ICCs: 0.95–0.99). The computed Cronbach’s alpha coefficients for domains of PSQ-39 were in the range good to excellent. The PSQ-39 showed good known-group validity and differentiated patients from the general population (Area under the curve [AUC] of 0.78 (95% CI: 0.73, 0.84). Construct validity evaluated by EFA led to extraction of seven factors (Cardiorespiratory, musculoskeletal, psychological, gastrointestinal, general, body balance and Globus), and the CFA confirmed the adequacy of extracted factors by EFA (CFI = 0.91, TLI = 0.90, PCFI = 0.77, PNFI = 0.71, CMIN = 1413.18 (df = 654), CMIN/DF = 2.16, and RMSEA = 0.06). Significant negative correlations between all domains of PSQ and SF-36 revealed an acceptable divergent Validity.

**Conclusions:**

The Persian version of the PSQ-39 is a reliable and valid questionnaire with applicability in a broad range of Persian language populations for assessing common psychosomatic symptoms in research as well as in clinical practice.

**Supplementary Information:**

The online version contains supplementary material available at 10.1186/s12888-021-03278-z.

## Background

Many primary care patients complain of physical symptoms that cannot be explained by an underlying organic disease/condition [1, 2]. These symptoms, so-called medically unexplained symptoms (MUS) or psychosomatic symptoms, are influenced by psychological conditions [2, 3]. While the majority of symptoms are self-resolved and mild, but some of them are severely disabling for the patient [1, 2]. Complaints such as headache, dizziness, fatigue, musculoskeletal pain, and gastrointestinal discomfort are prevalent in the general population; so that it is estimated that 80% of individuals experience one or more psychosomatic symptoms in a period of their life [4, 5]. Because these symptoms are associated with impaired quality of life, increased functional limitations, health-related job loss, and increased healthcare costs [2, 6], the assessment and recognition of psychosomatic symptoms’ burden are essential in both clinical care and research settings.

Physicians, researchers, and other healthcare professionals often assess the occurrence of psychosomatic symptoms by using self-report questionnaires [6, 7]. Such questionnaires present a supplementary source of information by capturing the patients’ perspectives of their symptoms [4]. Two systematic reviews [7, 8] indicated that there are different self-reported questionnaires for assessing psychosomatic symptoms, with the differences in a number and type of symptoms, length, scaling, dimensionality, reliability, validity, and studied populations (e.g. Cambodian Somatic Symptom and Syndrome Inventory (SSI) [9]; Patient Health Questionnaire (PHQ) [10]; Somatic Symptom Scale (SSS) [11]; Symptom Checklist-90 (SCL- 90) [12]; Brief Symptom Inventory (BSI) [13], and Bodily Distress Syndrome (BDS) [1]). While those brief self-report questionnaires that are mostly used for screening, are not common for measurement of psychosomatic symptom burden [11]. Some of these questionnaires are based on reporting life-time symptoms (e.g. the SSI); resulting the answers from patients are subjected to recall bias and underreporting of the items [6, 7]. Some other questionnaires inquire to report the symptoms within a week or a month (e.g., the PHQ-15 and SCL- 90R). Some of these questionnaires only ask questions about the presence or absence of symptoms, while others ask about symptoms severity [7]. In 2013, Lacourt et al. conducted a study in the Netherlands for clustering of functional somatic syndromes based on a 47-item psychosomatic symptoms questionnaire and the content of the questionnaire has been structured based on the Bodily Sensations Questionnaires [14, 15]. The questionnaire includes a wide range of symptoms including gastrointestinal, cardiac, respiratory, physical fatigue, musculoskeletal, cognitive, and other symptoms [14].

There are only three Iranian validated questionnaires for assessing psychosomatic symptoms including “Screening for somatic symptom disorders-7 (SOMS-7)”, “Patient health questionnaire (PHQ-15)” and “Somatic Symptom Scale (SSS)”, an abbreviated 8-item version of the PHQ-15 [16–18]. SOMS-7 is a questionnaire with 53 items designed to evaluate the effects of treatment in patients with somatic symptom disorders. The Persian version of the SOMS-7 has been validated in the general population and it has 47 items and 4 dimensions (pain, cardiovascular and respiratory symptoms), (gastrointestinal and urologic symptoms), (neurological functioning symptoms), (musculoskeletal symptoms) [16]. The items of PHQ-15 and SSS-8 assess the gastrointestinal problems, headaches, joint pain, dizziness, and difficulty falling asleep and are scored based on a 5-point Likert-type scale [17, 18]. The factor structure of both questionnaires reflects gastrointestinal problems, pain, fatigue, and cardiopulmonary domains for somatoform symptoms. The validation process of the PHQ-15 and SSS-8 was done in patients and university students, respectively not in the general population [17, 18]. Of the main limitations of these questionnaires are few items for evaluating the somatoform symptoms, and they assess symptoms during long retrospective periods. The present study aimed to culturally adapt and evaluate the psychometric properties a Persian version derived from the original 47-item psychosomatic symptoms’ questionnaire developed by in Lacourt et al. [14]. The number of items in Persian version of questionnaire was reduced to 39 in the validation process; therefore, it was named PSQ-39. Our validated questionnaire contains a large number of psychosomatic symptoms that assess the severity of symptoms in the last 7 days. In addition, its psychometric properties have been evaluated in a large sample size of the general adult population. We followed the guidelines for cross-cultural adaptation of questionnaires [19] to assess the psychometric properties of PSQ-39.

## Methods

### Study design and participants

This study was conducted between August 2018 and November 2018 among 996 Persian-speaking persons (896 healthy individuals and 100 patients) in Isfahan, the largest city in the central regions of Iran. The healthy individuals were selected from urban healthcare centres of Isfahan through multistage cluster random sampling. Isfahan city has 2 main leading and focal healthcare centres, i.e., the Isfahan focal healthcare centers I and II covering all local healthcare centers located at different geographic regions of Isfahan city. Focal healthcare centers I and II cover 23 and 22 local healthcare centers, respectively. We selected randomly 6 and 9 local healthcare centers from Isfahan healthcare centers I and II, respectively as the second-stage clusters based on considering the geographic coverage of the city for getting a representative sample. Then in each selected healthcare center, people who fulfilled our inclusion criteria were recruited based on convenience sampling. The inclusion criteria to the study were aged 18 years and over, able to read and write Persian, permanent resident of Isfahan city. The excluding criteria were as follows: being pregnant and affecting with major psychological and cognitive problems and physical illness at the time of participation in our study. Finally, 796 general adults agreed to participate in the study. The information of these participants was used to construct and divergent validities. The mean (SD) age was 40.07 (13.2) years. They consisted of 400 (50.3%) females and 659 (84.1%) married. About 43% of the study participants had college education, and 12.2% had adequate income. History of taking medication for psychological problems was reported by 117 (14.9%) of healthy individuals (Table 1). We selected 100 outpatients from people who attended different outpatient clinics including neurology, internal medicine, nephrology, gastroenterology, cardiology, rheumatology, and ear, nose, and throat (ENT) for preliminary clinical or diagnostic examinations in referral hospitals affiliated with Isfahan University of Medical Sciences (“Al-Zahra”, and “Noor and Hazrat-e-Ali Asghar”). The mean (SD) age was 47.89 (16.1) years. They consisted of 50 (50%) females and 83 (84.7%) married. About 23.2% of the outpatients had college education, and 2.1% had adequate income. History of taking medication for psychological problems was reported by 45% of them (Table 1). Based on self-report and checking the medical records of those patients who attended outpatients’ clinics we excluded people with confirmed chronic physical and mental diseases. Patients were aged over 18 years old, and those who non-Persian speaking, and did not consent to participate in our study were excluded. Two trained interviewers explained the purposes of the study to all eligible participants and then invited them to participate in the study. All participants received enough information about the study and also provided informed consent. The questionnaires were completed as a self-report. The design of the current study was approved by the Ethics Committee of Isfahan University of Medical Sciences (Project Number: 396963 and ethics approval code: IR.MUI.REC.1396.3.963).

**Table 1:** Participants characteristics by studied groups

		General people (***n*** = 796)	Patients (***n*** = 100)
**Age (years)**		40.07 ± 13.2	47.89 ± 16.1
**Sex**	Female	400 (50.3)	50 (50)
	Male	396 (49.7)	50 (50)
**Educational level**	Under Diploma (< 12 yrs)	214 (26.9)	57 (57.6)
	Diploma (12 yrs)	242 (30.4)	19 (19.2)
	Collegiate (> 12 yrs)	339 (42.7)	23 (23.2)
**Marital status**	Single	117 (14.9)	13 (13.3)
	Married	659 (84.1)	83 (84.7)
	Widow	8 (1.0)	2 (2.0)
**Income status**	Inadequate	165 (21.4)	48 (50)
	Middle	513 (66.5)	46 (47.9)
	Adequate	94 (12.2)	2 (2.1)
**History of taking medication for psychological problems**	No	668 (85.1)	55 (55.0)
	Yes	117 (14.9)	45 (45.0)
**History of taking medication for digestive diseases**	No	533 (68.3)	44 (44.0)
	Yes	247 (31.7)	56 (56.0)
**Smoking**	Nonsmoker	664 (84.3)	72 (72.0)
	Former smoker	48 (6.1)	13 (13.0)
	Current smoker	76 (9.6)	15 (15.0)
**Sleep duration (hour)**		7.68 ± 1.55	7.40 ± 1.54

Values are mean ± SD or frequency (percentage). The discrepancy in reported frequencies is related to availible missing data in some variables

### Procedures

#### The psychosomatic symptoms questionnaire

Lacourt et al. (2013) developed a questionnaire to measure psychosomatic symptoms. It comprised a list of 47 symptoms which primarily was based on the Bodily Sensations Questionnaires [14, 15]. Participants were asked ‘Have you been experienced’ the following symptom during the last week, symptoms such as pounding heart, chest pain, and shortness of breath, and each one has a five-point Likert scale ranging from 1 (not at all), 2 (a little), 3 (quite a bit), 4 (quite a lot), and 5 (highly). The questionnaire included *four gastrointestinal symptoms* (upset stomach, abdominal pain or stomach pain, bowel cramps, and bloated stomach), *six cardiac symptoms* (chest pain, rapid heart beat, pounding heart, tightness around the chest, irregular heartbeat, and painful stings in the heart area), *five respiratory symptoms* (feelings of dyspnea, shortness of breath, inability to take a deep breath, sudden fast or deep breathing, and breathlessness), *six physical fatigue symptoms* (feeling low on energy, feeling tired, feeling exhausted, feeling physically weak, not feeling fit, and feelings of muscle weakness), *six musculoskeletal symptoms* (muscle pain, pain in bones, pain in joints, back pain, pain in neck, and stiffness of fingers, arms, or legs), *six cognitive symptoms* (difficulty concentrating, forgetfulness, having trouble paying attention, unclear or foggy thoughts, distracting thoughts, confusion or feelings of unreality), *and 14 ‘other’ symptoms* (excessive sweating, hot or cold flashes, dry mouth, headache, trembling of hands, arms, or legs, tingling feeling in fingers, arms, or legs, numb feeling somewhere in body, nausea, fainting, having trouble swallowing, sore throat, rustling sound in ears, lump in throat, dizziness).

#### The 36-item short-form health survey (SF-36) questionnaire

The SF-36 questionnaire is a general quality of life questionnaire that measures eight components [20]. The first 4 components i.e., physical functioning (PF), role limitations due to physical health (RP), body pain (BP), and general health (GH) are used to calculate physical component and the last 4 components i.e., mental health (MH), role limitations due to emotional problems (RE), vitality (energy/fatigue) (VT), and social functioning (SF) are used to calculate mental component. SF-36 is scored from 0 to 100 and a higher score indicates a better quality of life. The psychometric properties of the Persian version of SF-36 had been evaluated previously. It showed satisfactory known group and convergent validity and also had acceptable internal consistency (Cronbach’s α = 0.65–0.90) [20].

In order to culturally adapt and evaluate the psychometric properties of the current study research tool, the following steps have been performed: 1) the translation of the questionnaire using the forward-backward method; 2) the evaluation of the face and content validity of the original version i.e. 47-item questionnaire developed by Lacourt et al. [14] and PSQ-39; 3) Evaluation of the construct validity of the PSQ-39 using EFA and CFA; 4) Evaluation of the divergent validity of the PSQ-39 in association with the SF-36; 5) Evaluation of the Known-groups validity of the PSQ; 6) Evaluation of the internal consistency and test-retest reliability of PSQ-39.

#### Translation

Permission was obtained from the developer (Tamara Lacourt, Utrecht, Netherlands), and the methodology recommended by Beaton et al. was followed to translate the questionnaire from English into the Persian language [19, 21]. In the forward stage, two translators translated items of the questionnaire into Persian. One of the translators was familiar with the concept of the questions being translated, but the second was unaware of the items being investigated in the original questionnaire. Then a unified version was established by the translators and presented to the study’s researchers (A.F., P.A., and H.R.). This final form then was backward translated into English by two other translators to compare with the original version based on conceptual balance. After a careful review by researchers (A.F., P.A., and H.R.) necessary changes were made and the provisional Persian version of the questionnaire was provided without any particular difficulty in it. Content validity was performed both qualitatively and quantitatively. In the qualitative phase, seven experts (two psychiatrists, one gastroenterologist, two internal medicine specialists, and two biostatisticians) carefully examined the items. In this phase those items with similar concepts or they had a substantial overlap in the 47-item version were merged, and finally a list of 39 symptoms was created which was named “psychosomatic symptoms questionnaire (PSQ-39)”. Items that were merged consisted of “rapid heartbeat/Pounding heart, feeling physically weak/feelings of muscle weakness, feelings of dyspnea/Shortness of breath, breathlessness/sudden fast or deep breathing, numb feeling somewhere in the body/Tingling feeling in fingers, arms, or legs, having trouble paying attention/difficulty concentrating, distracting thoughts/unclear or foggy thoughts and pain in joints/Pain in bones”. Consequently, we performed quantitative content validity by calculating the Content Validity Index (CVI) and Content Validity Ratio (CVR) for finalized PSQ-39. The expert panel evaluated the simplicity, relevancy, and clarity of each item, concerning the construct, on a 4-point rating scale. For example, the panel of experts assessed the simplicity of the items by using: (1) It’s complicated; (2) It needs serious revision; (3) It’s simple but it needs revision; and (4) It’s quite simple. A CVI of ≥ 0.79 was considered acceptable for each item [22]. The CVR evaluates the necessity of each item. For calculating CVR, seven experts were asked to rate the necessity of the PSQ-39 items on a three-point scale i.e., 1: unnecessary; 2: useful but unnecessary; and 3: necessary. A CVR of ≥ 0.99 was considered satisfactory for each item [23]. The qualitative face validity of PSQ-39 was examined by evaluating the feedback from a sample of healthy individuals aged more than 18 years; they evaluated the items for difficulty, relevancy, and ambiguity. We also asked them to check the importance of each item in the questionnaire. After these stages, the final Persian version of the PSQ-39 was developed and its psychometric properties have been evaluated.

### Validity

#### Construct validity

The factor structure of the PSQ-39 was explored using the EFA and CFA on the 796 general adults. According to the cross-validation method, we split our sample into two subsamples randomly. EFA was performed on the first half sample (training sample) based on the principal axis factoring extraction approach for estimating the factor loadings and the orthogonal Varimax rotation for interpretation of the extracted factors. We kept factors for further analysis based on the eigenvalues and Scree plot. We supposed factor-item loadings values greater than 0.40 and factors with eigenvalues > 1 as cutoff can result in more interpretable factors and explain sufficient amounts of the overall variation. Kaiser-Meyer-Olkin (KMO) measure of sample adequacy (Values > 0.7) and Bartlett’s Test of Sphericity (*P* < 0.05) were used to data viability for factorability [24]. According to the loaded items in each factor we labeled each extracted factor. We computed the score for each subscale (factor) by summing up items multiplied by related loading, and then we assigned the score to each participant. After that, we conducted a CFA on the validation sample to confirm the obtained factor structure from EFA. Parameters were estimated in CFA using the maximum likelihood method. Comparative Fit Index (CFI) ≥0.9, Tucker Lewis index (TLI) ≥0.9, Parsimony Comparative Fit Index (PCFI) > 0.5, Parsimony Normed Fit Index (PNFI) > 0.5, chi-square and degrees of freedom, Chi-square/ degree of freedom ratio < 3, and Root Mean Square Error of Approximation (RMSEA) < 0.08 were used to confirm the goodness of fit of the CFA [25].

#### Known-groups validity

‘Known-group’ validity describes the ability of a tool to distinguish between people with and without a health condition [26]. We assessed known-group validity based on the PSQ-39 ability to discriminate between healthy individuals and patients in terms of the prevalence of psychosomatic symptoms and the total score of the questionnaire. The validity of the measure is supported if the mean score and prevalence of the PSQ-39 items are significantly different between the two groups. We distributed the PSQ-39 questionnaire among 796 healthy individuals and 100 outpatients and compared their responses. We hypothesized that the score of PSQ-39 or prevalence of symptoms for these outpatients would be significantly higher than healthy people. We tested the difference in the distribution of answers to each item as well as the score of each subscale between two groups using the Chi-squared test and independent Student’s t-test, respectively. Also, Receiver Operating Characteristic Curve (ROC) was used to find the optimal cut-off value of the total score of the PSQ-39 questionnaire for discriminating healthy individuals from patients, the sensitivity and specificity along with the area under the curve (AUC) were reported.

#### Divergent validity

The Persian version of SF-36 questionnaire was used for evaluating divergent validity. We assessed the divergent validity of our questionnaire by calculating of Pearson correlation coefficient between the score of each PSQ-39 subscale and physical dimensions and the psychological dimensions of, respectively. We hypothesized a negative correlation between psychosomatic score and quality of life. This hypothesis for divergent validation was based on the fact that our questionnaire and SF-36 evaluate similar concepts however in opposite direction accordingly the scores of two questionnaires are in different directions. We also, calculated the Pearson correlation coefficient for each item and its domain (with the item removed) to examine item-scale correlations. According to Terwee et al.’s guidelines, item convergent validity should be at least 0.40 [27].

### Reliability & ceiling and floor effects

To investigate internal consistency and test-retest reliability, we recruited 100 healthy individuals aged 18 years old and over. The participants were asked to complete the PSQ-39 measure on two separate days with a 10 days interval. To evaluate test-retest reliability, the intraclass correlation coefficient (ICC) with 95% confidence using a two-way mixed model of absolute agreement type was estimated. We considered the ICC more than 0.70 was as excellent [27]. We also used Cronbach’s α coefficient to evaluate internal consistency with values between 0.70 to 0.95 as satisfactory [27]. Data collected in the first administration of the PSQ-39 measure (test-retest phase) was used to evaluate internal consistency and ceiling and floor effects. We also reported these indices based on the main sample (*n* = 896).

#### Other variables and statistical analysis

Additional data about age, sex, education level, marital status, income level, history of taking medication for psychological problems and digestive diseases, smoking, and sleep duration were also collected. In this paper, quantitative and qualitative variables were expressed as mean (SD) and frequency (percent), respectively. Holm-Bonferroni method [28] was used to adjust the type one error rate for multiple comparisons and adjusted *p*-value thresholds for significance were reported in Tables. R free statistical software version 3.2.2 and SPSS AMOS 16.0 (SPSS Inc., Chicago, IL, USA) were used for data analysis.

## Results

### Content and face validity

The experts’ committee checked the simplicity, relevancy, clarity, and necessity of the questionnaire’s items. The CVI ranged between 0.86 and 1.00, exceeded the acceptable threshold for all items. Also, the CVRs were 1.00 for all items. Consequently, no items were deleted (Table 2). A subsample of our study’s healthy participants checked the face validity of the finalized questionnaire. They stated that did not have any difficulties or ambiguities when completing the questionnaire’s items, also, they scored all items as important and relevant, so we did not change the content of the included symptoms in the questionnaire.

**Table 2:** Relevance, Simplicity, Clarity, Item Content Validity Index (I-CVI), and Content Validity Ratio (CVR) Values of the PSQ-39

Items	Relevance	Simplicity	Clarity	I-CVI	CVR
Headache	1	1	1	1	1
Dizziness	1	0.86	0.86	0.91	1
Fainting	1	0.86	0.86	0.91	1
Nausea	1	1	1	1	1
Rustling sound in ears	1	1	1	1	1
Confusion or feelings of unreality	1	0.86	0.86	0.91	1
Upset stomach	1	0.86	0.86	0.91	1
Abdominal pain	1	0.86	0.86	0.91	1
Bowel cramps	1	0.86	0.86	0.91	1
Bloated stomach	1	0.86	0.86	0.91	1
Feeling low on energy	1	0.86	0.86	0.91	1
Feeling tired	1	0.86	0.86	0.91	1
Feeling exhausted	1	0.86	0.86	0.91	1
Not feeling fit	1	0.86	0.86	0.91	1
Chest pain	1	0.86	0.86	0.91	1
Tightness around the chest	1	0.86	0.86	0.91	1
Irregular heart beat	1	0.86	0.86	0.91	1
Pounding heart	1	0.86	0.86	0.91	1
Painful stings in the heart area	1	0.86	0.86	0.91	1
Shortness of breath	1	1	1	1	1
Inability to take a deep breath	1	1	1	1	1
Sudden fast or deep breathing	1	0.86	0.86	0.91	1
Muscle pain	1	0.86	0.86	0.91	1
Pain in bones	1	0.86	0.86	0.91	1
Back pain	1	1	1	1	1
Pain in neck	1	1	1	1	1
Feelings of muscle weakness	1	1	1	1	1
Stiffness of fingers	1	1	1	1	1
Trembling of hands	1	1	1	1	1
Excessive sweating	1	1	1	1	1
Hot or cold flashes	1	1	1	1	1
Tingling feeling in fingers, arms, or legs	1	1	1	1	1
Dry mouth	1	1	1	1	1
Lump in throat	1	1	1	1	1
Having trouble swallowing	1	0.86	0.86	0.91	1
Sore throat	1	1	1	1	1
Forgetfulness	1	0.86	0.86	0.91	1
Difficulty concentrating	1	0.86	0.86	0.91	1
Unclear or foggy thoughts	1	0.86	0.86	0.91	1

### Construct validity

**Fig. 1:**
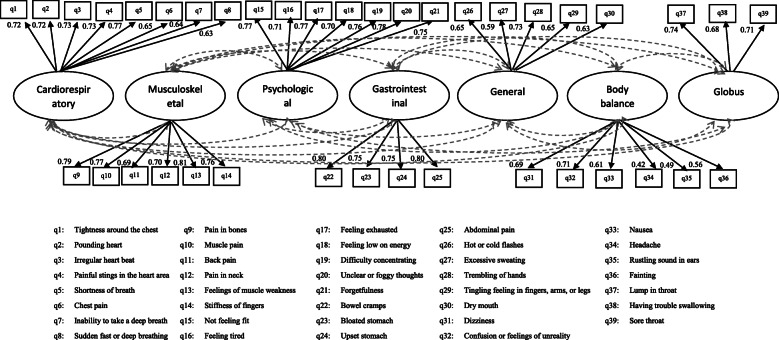
Confirmatory factor analysis testing the extracted construct from EFA on the PSQ-39 items

EFA with Varimax rotation extracted seven factors from the PSQ-39 measure which were labeled as “cardiorespiratory”, “musculoskeletal”, “psychological”, “gastrointestinal”, “general”, “body balance” and “Globus” accounting for 13.1, 10.1, 9.8, 7.6, 7.6, 6.7, and 6.4% of total variance, respectively. A KMO value of 0.91 and *P* < 0.05 for the Bartlett’s test confirmed the sample size adequacy and data factorability, respectively. Table 3 provides the factor loadings of seven extracted factors from EFA on the 39 items of the PSQ-39 measure. The results obtained from the CFA indicated a good fit according to goodness of fit indices as follows: CFI = 0.91, TLI = 0.90, PCFI = 0.77, PNFI = 0.71, CMIN = 1413.17 (df = 654), CMIN/DF = 2.16, and RMSEA = 0.06 were confirmed goodness of fit of factor model also all items loaded significantly on their respective factors (Fig. 1).

**Table 3:** Factor loadings and item-scale correlations of PSQ-39 items

Psychosomatic symptoms	Extracted factors ^**a**^							
	Cardiorespiratory	Musculoskeletal	Psychological	Gastrointestinal	General	Body balance	Globus	Item-scale correlations^**^
Tightness around the chest	***0.79***	0.12	0.18	0.07	−0.02	0.14	0.14	0.74
Pounding heart	***0.76***	0.08	0.18	0.09	0.16	0.16	0.10	0.74
Irregular heart beat	***0.75***	0.08	0.20	0.08	0.14	0.17	0.12	0.74
Painful stings in the heart area	***0.72***	0.13	0.16	0.13	0.09	0.05	0.26	0.72
Shortness of breath	***0.70***	0.16	0.15	0.10	0.26	0.16	0.12	0.74
Chest pain	***0.70***	0.21	0.20	0.14	−0.02	0.09	0.18	0.69
Inability to take a deep breath	***0.57***	0.23	0.08	0.08	0.37	0.12	0.12	0.60
Sudden fast or deep breathing	***0.56***	0.24	0.03	0.11	0.41	0.17	0.04	0.63
Pain in bones	0.24	***0.75***	0.19	0.09	0.22	0.10	0.04	0.78
Muscle pain	0.30	***0.73***	0.15	0.16	0.17	0.12	0.00	0.76
Back pain	0.07	***0.68***	0.16	0.20	0.16	0.10	0.14	0.67
Pain in neck	0.06	***0.67***	0.08	0.11	0.16	0.11	0.24	0.63
Feelings of muscle weakness	0.14	***0.64***	0.37	0.10	0.22	0.12	0.17	0.73
Stiffness of fingers	0.19	***0.58***	0.21	0.10	0.31	−0.01	0.14	0.64
Not feeling fit	0.31	0.24	***0.72***	0.17	0.07	0.14	0.06	0.75
Feeling tired	0.24	0.29	***0.68***	0.28	0.04	0.20	−0.04	0.72
Feeling exhausted	0.33	0.29	***0.66***	0.13	0.15	0.17	0.02	0.73
Feeling low on energy	0.23	0.27	***0.64***	0.31	0.11	0.17	−0.04	0.69
Difficulty concentrating	0.14	0.13	***0.62***	0.08	0.33	0.17	0.40	0.73
Unclear or foggy thoughts	0.18	0.13	***0.60***	0.10	0.34	0.15	0.38	0.73
Forgetfulness	0.14	0.19	***0.55***	0.09	0.34	0.11	0.39	0.67
Bowel cramps	0.15	0.13	0.15	***0.78***	0.06	0.05	0.17	0.68
Bloated stomach	0.15	0.19	0.18	***0.77***	0.19	0.08	0.06	0.68
Upset stomach	0.06	0.12	0.14	***0.77***	0.12	0.22	0.09	0.70
Abdominal pain	0.13	0.13	0.14	***0.71***	0.04	0.28	0.09	0.66
Hot or cold flashes	0.14	0.22	0.15	0.13	***0.72***	0.04	0.05	0.64
Excessive sweating	0.15	0.24	0.18	0.16	***0.70***	0.02	0.02	0.62
Trembling of hands	0.29	0.29	0.21	0.00	***0.48***	0.09	0.22	0.56
Tingling feeling in fingers, arms, or legs	0.12	0.42	0.18	0.08	***0.46***	0.07	0.09	0.55
Dry mouth	0.15	0.28	0.10	0.10	***0.43***	0.16	0.23	0.49
Dizziness	0.19	0.23	0.16	0.10	−0.05	***0.72***	0.08	0.64
Confusion or feelings of unreality	0.05	−0.02	0.32	−0.03	0.22	***0.63***	0.14	0.48
Nausea	0.22	0.12	0.00	0.28	0.03	***0.62***	0.02	0.52
Headache	0.11	0.37	0.13	0.16	−0.18	***0.53***	−0.03	0.42
Rustling sound in ears	0.12	0.03	0.06	0.16	0.19	***0.51***	0.03	0.40
Fainting	0.31	−0.11	0.22	0.09	0.10	***0.42***	0.26	0.40
Lump in throat	0.24	0.12	0.07	0.12	0.21	0.04	***0.74***	0.62
Having trouble swallowing	0.34	0.15	0.08	0.13	−0.07	0.01	***0.72***	0.54
Sore throat	0.15	0.19	0.10	0.11	0.12	0.17	***0.65***	0.51
Variance explained* (%)	13.1	10.1	9.8	7.6	7.6	6.7	6.4	–

^a^ Exploratory factor analysis with Varimax rotation; Factor loadings < 0.4 are not shown for simplicity; * Variance explained resulted from factor analysis; ** Pearson’s correlation coefficients between each item and its own domain (extracted factors) corrected for overlap;

All item-scale correlations based on Pearson’s correlation coefficients exceeded the set value of 0.40 (Table 3). 

### Known-groups validity

**Fig. 2:**
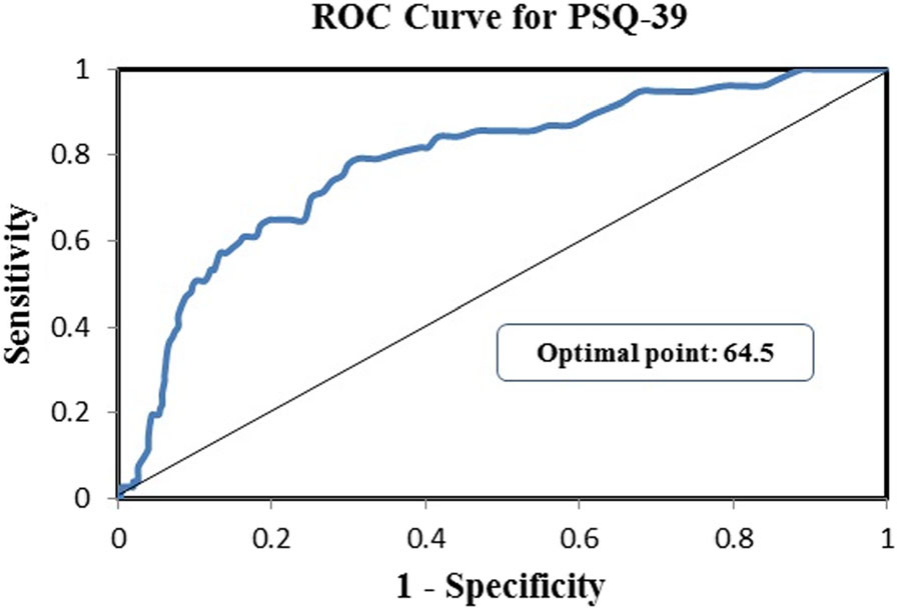
Receiver - Operator Characteristic (ROC) Curve for the total score of PSQ-39 questionnaire as related to studied groups (healthy individuals and patients)

Table 4 provides the distribution of each psychosomatic symptom in two studied groups (healthy individuals and patients). The majority of psychosomatic symptoms, except for back pain, bowel cramps, abdominal pain, and sore throat significantly more prevalent in the outpatients’ group (*p* < 0.001; Table 4). In addition, the comparison of the mean (SD) of the PSQ-39 subscales between studied groups is presented in Table 5. As expected, the mean (SD) of all the extracted subscales of the PSQ-39 was significantly higher in outpatients (*p* < 0.01; Table 5). The Known-groups validity of the PSQ-39 was evaluated based on the value of the total score of the tool for differentiating outpatients from healthy group. The ROC curve was generated and results showed strong evidence about the accuracy of our study validated questionnaire for discriminating outpatients from healthy individuals with an area under the curve [AUC] of 0.79 (95% CI: 0.73, 0.84, *p* < 0.001) and optimal cutoff point 64.5 (sensitivity, 80%, and specificity, 69%) (Fig. 2).

**Table 4:** Distribution of Psychosomatic symptoms in studied groups

Psychosomatic symptoms	General					patients					***P*** value*	Significant level**
	Not	A Little	Quite A Bit	Quite A Lot	Highly	Not	A Little	Quite A Bit	Quite A Lot	Highly		
Tightness around the chest	632 (80)	94 (11.9)	45 (5.7)	14 (1.8)	5 (0.6)	48 (48.5)	18 (18.2)	21 (21.2)	11 (11.1)	1 (1.0)	< 0.0001	0.0013
Pounding heart	588 (74.1)	111 (14)	66 (8.3)	23 (2.9)	5 (0.6)	40 (40.4)	20 (20.2)	28 (28.3)	9 (9.1)	2 (2.0)	< 0.0001	0.0013
Irregular heart beat	592 (74.8)	109 (13.8)	66 (8.3)	19 (2.4)	5 (0.6)	39 (39.4)	25 (25.3)	22 (22.2)	11 (11.1)	2 (2.0)	< 0.0001	0.0014
Painful stings in the heart area	598 (75.9)	116 (14.7)	48 (6.1)	20 (2.5)	6 (0.8)	52 (52.5)	17 (17.2)	16 (16.2)	13 (13.1)	1 (1.0)	< 0.0001	0.0014
Shortness of breath	537 (67.9)	157 (19.8)	62 (7.8)	27 (3.4)	8 (1.0)	34 (34)	24 (24)	25 (25)	15 (15.0)	2 (2.0)	< 0.0001	0.0014
Chest pain	611 (77.3)	103 (13)	56 (7.1)	15 (1.9)	5 (0.6)	40 (40.4)	19 (19.2)	26 (26.3)	12 (12.1)	2 (2.0)	< 0.0001	0.0015
Inability to take a deep breath	561 (71.5)	148 (18.9)	57 (7.3)	15 (1.9)	4 (0.5)	38 (38.8)	32 (32.7)	22 (22.4)	5 (5.1)	1 (1.0)	< 0.0001	0.0015
Sudden fast or deep breathing	508 (64.6)	189 (24)	68 (8.7)	19 (2.4)	2 (0.3)	33 (33.3)	31 (31.3)	25 (25.3)	9 (9.1)	1 (1.0)	< 0.0001	0.0016
Pain in bones	384 (48.7)	187 (23.7)	134 (17)	54 (6.9)	29 (3.7)	31 (31)	20 (20)	24 (24)	18 (18.0)	7 (7.0)	< 0.0001	0.0016
Muscle pain	370 (47.1)	200 (25.5)	141 (18)	56 (7.1)	18 (2.3)	27 (27.6)	26 (26.5)	25 (25.5)	14 (14.3)	6 (6.1)	< 0.0001	0.0017
Back pain	333 (42.3)	200 (25.4)	155 (19.7)	68 (8.6)	32 (4.1)	32 (32.3)	24 (24.2)	28 (28.3)	11 (11.1)	4 (4.0)	0.205	0.0250
Pain in neck	493 (62.6)	149 (18.9)	82 (10.4)	43 (5.5)	20 (2.5)	40 (40.4)	23 (23.2)	21 (21.2)	12 (12.1)	3 (3.0)	< 0.0001	0.0017
Feelings of muscle weakness	401 (50.8)	202 (25.6)	108 (13.7)	59 (7.5)	19 (2.4)	28 (28)	19 (19)	21 (21)	30 (30.0)	2 (2.0)	< 0.0001	0.0018
Stiffness of fingers	561 (71.2)	111 (14.1)	73 (9.3)	33 (4.2)	10 (1.3)	42 (42)	20 (20)	28 (28)	9 (9.0)	1 (1.0)	< 0.0001	0.0019
Not feeling fit	277 (34.9)	244 (30.8)	165 (20.8)	81 (10.2)	26 (3.3)	19 (19.2)	21 (21.2)	22 (22.2)	25 (25.3)	12 (12.1)	< 0.0001	0.0019
Feeling tired	237 (30)	215 (27.2)	196 (24.8)	114 (14.4)	28 (3.5)	13 (13)	14 (14)	33 (33)	30 (30.0)	10 (10.0)	< 0.0001	0.0020
Feeling exhausted	405 (51.5)	171 (21.7)	124 (15.8)	58 (7.4)	29 (3.7)	24 (24)	15 (15)	24 (24)	24 (24.0)	13 (13.0)	< 0.0001	0.0021
Feeling low on energy	337 (42.7)	195 (24.7)	165 (20.9)	78 (9.9)	15 (1.9)	16 (16.2)	21 (21.2)	26 (26.3)	27 (27.3)	9 (9.1)	< 0.0001	0.0022
Difficulty concentrating	411 (51.9)	214 (27)	93 (11.7)	50 (6.3)	24 (3.0)	26 (26)	21 (21)	31 (31)	17 (17.0)	5 (5.0)	< 0.0001	0.0023
Unclear or foggy thoughts	470 (59.3)	152 (19.2)	93 (11.7)	45 (5.7)	32 (4.0)	28 (28.6)	21 (21.4)	27 (27.6)	16 (16.3)	6 (6.1)	< 0.0001	0.0024
Forgetfulness	397 (50.1)	218 (27.5)	109 (13.8)	50 (6.3)	18 (2.3)	25 (25)	19 (19)	26 (26)	21 (21.0)	9 (9.0)	< 0.0001	0.0025
Bowel cramps	535 (67.7)	158 (20)	72 (9.1)	21 (2.7)	4 (0.5)	59 (61.5)	24 (25)	10 (10.4)	3 (3.1)	0 (0.0)	0.675	0.0500
Bloated stomach	476 (60.3)	155 (19.6)	105 (13.3)	40 (5.1)	14 (1.8)	40 (40.8)	30 (30.6)	18 (18.4)	9 (9.2)	1 (1.0)	.004	0.0071
Upset stomach	448 (56.5)	181 (22.8)	117 (14.8)	38 (4.8)	9 (1.1)	31 (31)	36 (36)	23 (23)	8 (8.0)	2 (2.0)	< 0.0001	0.0026
Abdominal pain	524 (66.8)	161 (20.5)	76 (9.7)	16 (2.0)	8 (1.0)	51 (52.6)	26 (26.8)	15 (15.5)	4 (4.1)	1 (1.0)	.068	0.0167
Hot or cold flashes	598 (76.1)	112 (14.2)	50 (6.4)	17 (2.2)	9 (1.1)	38 (38)	25 (25)	31 (31)	5 (5.0)	1 (1.0)	< 0.0001	0.0028
Excessive sweating	556 (70.3)	119 (15)	66 (8.3)	31 (3.9)	19 (2.4)	41 (41.8)	14 (14.3)	16 (16.3)	25 (25.5)	2 (2.0)	< 0.0001	0.0029
Trembling of hands	609 (77.1)	112 (14.2)	47 (5.9)	14 (1.8)	8 (1.0)	40 (40.4)	33 (33.3)	15 (15.2)	8 (8.1)	3 (3.0)	< 0.0001	0.0031
Tingling feeling in fingers, arms, or legs	541 (68.3)	145 (18.3)	66 (8.3)	27 (3.4)	13 (1.6)	43 (43)	32 (32)	17 (17)	6 (6.0)	2 (2.0)	< 0.0001	0.0033
Dry mouth	543 (68.7)	172 (21.8)	50 (6.3)	19 (2.4)	6 (0.8)	38 (38)	28 (28)	18 (18)	15 (15.0)	1 (1.0)	< 0.0001	0.0036
Dizziness	519 (65.3)	152 (19.1)	101 (12.7)	20 (2.5)	3 (0.4)	44 (44.4)	25 (25.3)	21 (21.2)	7 (7.1)	2 (2.0)	< 0.0001	0.0038
Confusion or feelings of unreality	664 (83.5)	81 (10.2)	41 (5.2)	7 (0.9)	2 (0.3)	54 (55.7)	20 (20.6)	14 (14.4)	9 (9.3)	0 (0.0)	< 0.0001	0.0042
Nausea	656 (82.8)	96 (12.1)	34 (4.3)	5 (0.6)	1 (0.1)	62 (62)	18 (18)	15 (15)	4 (4.0)	1 (1.0)	< 0.0001	0.0045
Headache	308 (39.2)	243 (30.9)	178 (22.6)	52 (6.6)	5 (0.6)	24 (24)	25 (25)	36 (36)	13 (13.0)	2 (2.0)	< 0.0001	0.0050
Rustling sound in ears	624 (78.7)	103 (13)	45 (5.7)	14 (1.8)	7 (0.9)	53 (53.5)	17 (17.2)	19 (19.2)	6 (6.1)	4 (4.0)	< 0.0001	0.0056
Fainting	701 (88.4)	56 (7.1)	26 (3.3)	7 (0.9)	3 (0.4)	75 (77.3)	13 (13.4)	6 (6.2)	3 (3.1)	0 (0.0)	.017	0.0083
Lump in throat	687 (87.5)	60 (7.6)	27 (3.4)	7 (0.9)	4 (0.5)	69 (69.7)	15 (15.2)	12 (12.1)	2 (2.0)	1 (1.0)	< 0.0001	0.0063
Having trouble swallowing	725 (91.7)	42 (5.3)	17 (2.1)	5 (0.6)	2 (0.3)	83 (83)	13 (13)	3 (3)	1 (1.0)	0 (0.0)	.040	0.0100
Sore throat	624 (78.9)	111 (14)	42 (5.3)	13 (1.6)	1 (0.1)	72 (72)	14 (14)	13 (13)	1 (1.0)	0 (0.0)	.067	0.0125

* *P* value from Pearson χ^2^. Values are frequency (percent). ** All *p*-values were adjusted using the false discovery rate controlling procedure developed by Holm-Bonferroni method

**Table 5:** Comparison of scores of the PSQ-39 subscales by studied groups

	General	Patients	***P***-Value	Significant level**
**Total Score**	60.31 (19.70)	83.69 (22.60)	< 0.001	0.006
**Cardiorespiratory**	11.21 (4.83)	16.63 (6.77)	< 0.001	0.007
**Musculoskeletal**	10.84 (5.06)	14.02 (5.17)	< 0.001	0.008
**Psychological**	13.82 (6.17)	19.48 (7.17)	< 0.001	0.010
**General**	7.20 (3.10)	10.33 (3.74)	< 0.001	0.013
**Body Balance**	8.46 (2.80)	11.09 (3.96)	< 0.001	0.017
**Globus**	3.60 (1.36)	4.15 (1.71)	0.003	0.025
**Gastrointestinal**	6.33 (2.94)	7.29 (2.75)	0.003	0.050
Area under the curve [AUC] (95% CI)*		0.786 (0.732, 0.840)		

Values are Mean (SD). P-Values are based on independent Student’s t-test. *AUC based on the total score of the PSQ-39

**Significant at *P* < 0.001, adjustment was made using the false discovery rate controlling procedure developed by Holm-Bonferroni method

### Divergent validity

PSQ-39 had moderate to high negative correlations with dimensions of the SF-36 ranging from − 0.70 to − 0.25 (*p* < 0.01) (Table 6). For example, the total score of PSQ-39 was inversely correlated with the physical (r = − 0.69, *p* < 0.001) and psychological components (r = − 0.66, *p* < 0.001) of the SF-36.

**Table 6:** Correlations to assess the divergent validity of the PSQ-39 vs. SF-36

	Cardiorespiratory	Musculoskeletal	Psychological	Gastrointestinal	General	Body Balance	Globus	Total score
**Physical functioning**	− 0.40	− 0.46	− 0.34	− 0.25	− 0.35	− 0.23	− 0.25	− 0.45
**Role functioning/physical**	− 0.38	− 0.49	− 0.48	− 0.30	− 0.39	− 0.34	− 0.25	− 0.52
**Role functioning/emotional**	−0.34	− 0.44	− 0.53	−0.32	− 0.39	−0.38	− 0.27	−0.50
**Vitality**	−0.46	−0.47	− 0.65	−0.38	− 0.42	−0.45	− 0.30	−0.60
**Emotional well-being**	−0.43	−0.42	− 0.57	−0.34	− 0.39	−0.44	− 0.29	−0.55
**Social functioning**	−0.46	−0.47	− 0.62	−0.40	− 0.43	−0.44	− 0.30	−0.61
**Bodily pain**	−0.49	−0.67	− 0.56	−0.44	− 0.48	−0.43	− 0.33	−0.66
**General health**	−0.44	− 0.48	− 0.51	−0.38	− 0.36	−0.40	− 0.32	−0.56
**Physical component**	−0.53	− 0.65	− 0.59	− 0.43	−0.50	− 0.43	−0.36	− 0.69
**Mental component**	−0.50	− 0.54	−0.70	− 0.43	−0.49	− 0.51	−0.35	− 0.66

All correlations are significant based on *p*-values adjusted using the false discovery rate controlling procedure developed by Holm-Bonferroni method

### Reliability analyses & ceiling and floor effects

The reliability and descriptive statistics for the seven PSQ-39 subscales are shown in Table 7. The ICC coefficient for the total score of the PSQ-39 suggests strong test-retest reliability (ICC = 0.99, 95% CI: 0.995 to 0.998; *p* < 0.001). The ICC coefficients for subscales “cardiorespiratory”, “musculoskeletal”, “psychological”, “gastrointestinal” and “general” were estimated to be 0.99 and for “body balance” and “Globus” subscales were 0.95 and 0.94, respectively, all suggest strong test-retest reliability. In addition, Cronbach’s alpha coefficient to indicate item internal consistency for each subscale is presented in Table 7. Cronbach’s alpha coefficient was calculated in both the internal pilot sample (*n* = 100) and total sample (*n* = 896). All scales met or exceeded the 0.70 level recommended except “body balance”. However, the Cronbach’s alpha coefficient in the original sample for this dimension is equal to 0.74, which is acceptable. In addition, the percentage of respondents scoring at the lowest level (i.e., floor effect) was substantial for subscales of “globus”, “general”, “cardiorespiratory”, and “gastrointestinal”. In contrast, the percentage of participants scoring at the highest level (i.e., ceiling effect) was minimal for all subscales.

**Table 7:** Descriptive statistics and reliability statistics for the PSQ-39 scales

	Mean (SD)		Cronbach’s α		ICC (%95CI)	Floor (%)		Ceiling (%)	
	***n*** = 896^1^	***n*** = 100^2^	***n*** = 896^1^	***n*** = 100^2^	***n*** = 100^2^	***n*** = 896^1^	***n*** = 100^2^	***n*** = 896	***n*** = 100^2^
**Cardiorespiratory**	11.8 (5.35)	11.75 (4.63)	0.92	0.88	0.986 (0.980, 0.991)	38.7	34.3	0.1	1
**Musculoskeletal**	11.2 (5.17)	10.76 (4.57)	0.88	0.87	0.993(0.989, 0.995)	23.6	25	0.1	1
**Psychological**	14.45 (6.53)	13.47 (5.41)	0.91	0.88	0.994 (0.990, 0.996)	16.3	16	0.6	1
**Gastrointestinal**	6.43 (2.94)	6.40 (2.74)	0.83	0.82	0.985 (0.978, 0.990)	38.6	31.3	0.2	1
**General**	7.55 (3.33)	7.55 (3.61)	0.80	0.85	0.989 (0.984, 0.993)	40.7	39	0.1	1
**Body Balance**	8.74 (3.06)	8.67 (2.61)	0.74	0.59	0.952 (0.930, 0.968)	28.4	21.2	0.1	1
**Globus**	3.66 (1.42)	3.61 (1.28)	0.72	0.70	0.945 (0.920, 0.963)	71.7	73	0.2	1
**Total Score**	62.74 (21.23)	61.90 (18.34)	0.95	0.94	0.997 (0.995,0.998)	8.2	2.1	0.1	1

*ICC* Intraclass Coefficient; ^1^ Total sample, ^2^ Pilot sample

## Discussion

In the current study, the psychometric properties of the Persian version of PSQ-39 were evaluated. The results of this study showed that the Persian version of PSQ-39 has excellent test-retest reliability and internal consistency. Patients had experienced higher levels of psychosomatic symptoms than healthy individuals, indicating good known-group validity. Construct validity of PSQ-339 was explored by exploratory factor analysis and well confirmed by applying confirmatory factor analysis. The questionnaire also showed satisfactory divergent validity based on association analysis with SF-36.

Reliability in the current study was assessed by the intraclass correlation (ICC) coefficient and Cronbach’s α. All subscales’ ICC coefficients exceeded 0.9, and approximately all Cronbach’s α coefficients were between 0.7 to 1, accordingly, the investigated questionnaire had strong test-retest reliability and internal consistency. PSQ-39 showed higher test-retest reliability than SOMS-7 (ICC of 0.86) and other questionnaires assessing somatoform symptoms in the Iranian population i.e. PHQ-15 and SSS-8 did not report this reliability measure [16–18]. The PSQ-39 questionnaire in the present study showed acceptable internal consistency nearly at the same levels which were observed in the previous works in Iran using other questionnaires [16–18].

According to our results, the Persian version of the PSQ-39 questionnaire well discriminated healthy individuals from outpatients; in which the prevalence and mean score of psychosomatic symptoms were significantly higher in patients. Also, a known-group analysis using ROC curve was conducted to determine an optimal cut-off point for the score of PSQ-39 with the highest sensitivity and specificity. These findings indicated that the score 64.5 has a sensitivity 80% and a specificity 69%. This result indicated acceptable known-group validity for this questionnaire. The result of Ebrahimi et al.’s study using the SOMS-7 questionnaire was similar to our study (Score of 15.5 as a cut-off point with a sensitivity of 77% and specificity of 66%) [16].

Applying factor analysis for evaluating construct validity resulted in seven factors (cardiorespiratory, musculoskeletal, psychological, gastrointestinal, general, body balance, and Globus). One of our limitations in the present study is that the psychometric properties of the original version of the questionnaire have not been evaluated. On the other hand, the number of items in the original version is different from the Persian version. Therefore, we used a cross-validation method to evaluate the construct validity. Results of CFA confirmed the adequacy of the extracted construct from EFA. Lacourt et al.’s study identified a pattern of gastrointestinal, cardiac, respiratory, physical fatigue, musculoskeletal, cognitive, and ‘other’ factors [14]. The dimensionality of other psychosomatic symptoms questionnaires has been investigated in previous studies, although they are not the same as our validated questionnaire however we compared the dimensions of the PSQ-39 with these questionnaires [1, 11, 14, 16, 29–31] in terms of number, constructive items and concept. Ebrahimi et al.’s study [16] in Iran indicated four factors using EFA (“pain and cardiorespiratory”, “gastrointestinal”, “neurological”, and “musculoskeletal”). Heidari et al. reported four factors (‘psycho-fatigue’, ‘gastrointestinal’, ‘neuro- skeletal’ and ‘pharyngeal-respiratory’) using factor mixture model [31]. Fink et al.’s study on 978 patients identified a pattern of cardiopulmonary (CP), musculoskeletal/pain (MS), and gastrointestinal (GI) factors [32]. The factor analyses in the Budtz-Lilly et al’ s study identified four distinct factors including cardiopulmonary, gastrointestinal, musculoskeletal, and general symptoms [1]. As was expected, each questionnaire has its dimensions different from other tools. These differences can be justified by the number and nature of symptoms/items, and more important the dependency of initiation and experience of psychosomatic symptoms to early and recent Life Events, chronic stress and allostatic load, health attitudes and behavior, social support, psychological well-being, personality factors, psychiatric disturbances and psychological symptoms [33] that these effective factors are socio-cultural dependent. In addition, most of the previous studies were conducted in clinical settings on a highly selective population.

### Study strengths and limitations

The major strength of the current study is that we investigated the wide variety of psychometric properties of the PSQ-39 questionnaire in a large sample of general Persian language people. In the present study the symptoms were also evaluated in the most recent time of their occurrence from the participants; therefore, this may reduce the risk of recall bias. Despite these strengths, this study is not without limitations. We selected the sample only from Isfahan (located at central region of Iran); therefore, the representativeness of this sample for all Iranian populations or other Persian language countries should be interpreted with susceptibility. The identified clusters of somatoform symptoms, based on factor analysis, in our study is not completely similar to the 47-item original version, the possible explainable reasons are: different statistical methods used and more importantly the psychometric properties of the original 47-item questionnaire have not been evaluated by its developers. Another limitation of current study that should be addressed is: To evaluate the known-group validity, we did not access to a group of patients with a definite diagnosis of psychosomatic disorders versus healthy individuals.

## Conclusions

The findings suggest that the Persian version of the psychosomatic symptoms’ questionnaire (PSQ-39) could produce reliable and valid measurements of psychosomatic symptoms in the Persian-speaking adult population. The PSQ-39 is self-report and easy to understand and takes nearly 20 min to be completed.

## Supplementary Information

**Additional file 1:.** Psychosomatic Symptoms Questionnaire (PSQ-39) in Persian.

**Additional file 2:.**

**Additional file 3:.**

## Data Availability

All data generated or analyzed during this study are included in this article (as a supplementary file in SPSS format).

## Abbreviations

PSQ: Psychosomatic Symptoms Questionnaire
ICC: Intraclass Correlation
EFA: Exploratory factor analysis
CFA: Confirmatory factor analysis
AUC: Area under the curve
ROC: Receiver Operating Characteristic
MUS: Medically unexplained symptoms
SSI: Cambodian Somatic Symptom and Syndrome Inventory
SSS: Somatic Symptom Scale
SCL-90: Symptom Checklist-90
BSI: Brief Symptom Inventory
BDS: Bodily Distress Syndrome
SOMS-7: Somatic symptom disorders-7
PHQ: Patient Health Questionnaire
ENT: Ear, nose, and throat
KMO: Kaiser-Meyer-Olkin
CFI: Comparative Fit Index
PCFI: Parsimony Comparative Fit Index
PNFI: Parsimony Normed Fit Index
RMSEA: Root Mean Square Error of Approximation
SF-36: 36-Item Short-Form Health Survey
PF: Physical functioning
RP: Role limitations due to physical health
BP: Body pain
GH: General health
MH: Mental health
RE: Role limitations due to emotional problems
VT: Vitality (energy/fatigue)
SF: Social functioning
CI: Confidence Interval
