# Publisher Correction to: Psychosomatic symptoms questionnaire (PSQ-39): a psychometric study among general population of Iranian adults

**DOI:** 10.1186/s12888-021-03328-6

**Published:** 2021-07-09

**Authors:** Zahra Heidari, Awat Feizi, Sara Rezaei, Hamidreza Roohafza, Peyman Adibi

**Affiliations:** 1 Department of Biostatistics and Epidemiology, School of Health, Isfahan University of Medical Sciences, P.O. Box 319, Hezar-Jerib Ave, Isfahan, 8174673461 Iran; 2 Cardiac Rehabilitation Research Center, Cardiovascular Research Institute, Isfahan University of Medical Sciences, Isfahan, Iran; 3 Student Research Committee, School of Health, Isfahan University of Medical Sciences, Isfahan, Iran; 4 Gastroenterology and Hepatology Research Center, Isfahan University of Medical Sciences, Isfahan, Iran; 5 Department of Internal Medicine, School of Medicine, Isfahan University of Medical Sciences, Isfahan, Iran

**Publisher Correction to: BMC Psychiatry 21, 269 (2021)**

**https://doi.org/10.1186/s12888-021-03278-z**

Following the publication of the original article [1], the authors identified that the incorrect additional file 1 was published. The correct file has been published.

The original article [1] has been corrected.

The publisher apologizes to the authors and readers for the inconvenience.

## Supplementary Information

**Additional file 1.** Psychosomatic Symptoms Questionnaire (PSQ-39).

## References

[1] Heidari (2021). Psychosomatic symptoms questionnaire (PSQ-39): a psychometric study among general population of Iranian adults. BMC Psychiatry.

