# Supplementary material for: Psychosomatic symptoms questionnaire (PSQ-39): a psychometric study among general population of Iranian adults
Source: BMC Psychiatry. 2021 May 25;21:269. doi: 10.1186/s12888-021-03278-z (PMC8147044; doi:10.1186/s12888-021-03278-z)
Supplement: Supplementary file 1 — Additional file 1:. Psychosomatic Symptoms Questionnaire (PSQ-39) in Persian. [file 12888_2021_3278_MOESM1_ESM.docx]

**Supplementary file:** Psychosomatic Symptoms Questionnaire (PSQ-39)

In this questionnaire, you are asked to what extent you were experiencing somatic symptoms. Please indicate **for every symptom in the list** the answer that is most applicable for you. For every symptom, we want to know how you felt during **the last week, including today**.

To what extend did you experience:

| Symptoms | Not at all | A bit | Quite a bit | Quite a lot | Highly |
| --- | --- | --- | --- | --- | --- |
| 1. Tightness around the chest |  |  |  |  |  |
| 1. Pounding heart |  |  |  |  |  |
| 1. Irregular heart beat |  |  |  |  |  |
| 1. Painful stings in the heart area |  |  |  |  |  |
| 1. Shortness of breath |  |  |  |  |  |
| 1. Chest pain |  |  |  |  |  |
| 1. Inability to take a deep breath |  |  |  |  |  |
| 1. Sudden fast or deep breathing |  |  |  |  |  |
| 1. Pain in bones |  |  |  |  |  |
| 1. Muscle pain |  |  |  |  |  |
| 1. Back pain |  |  |  |  |  |
| 1. Pain in neck |  |  |  |  |  |
| 1. Feelings of muscle weakness |  |  |  |  |  |
| 1. Stiffness of fingers |  |  |  |  |  |
| 1. Not feeling fit |  |  |  |  |  |
| 1. Feeling tired |  |  |  |  |  |
| 1. Feeling exhausted |  |  |  |  |  |
| 1. Feeling low on energy |  |  |  |  |  |
| 1. Difficulty concentrating |  |  |  |  |  |
| 1. Unclear or foggy thoughts |  |  |  |  |  |
| 1. Forgetfulness |  |  |  |  |  |
| 1. Bowel cramps |  |  |  |  |  |
| 1. Bloated stomach |  |  |  |  |  |
| 1. Upset stomach |  |  |  |  |  |
| 1. Abdominal pain |  |  |  |  |  |
| 1. Hot or cold flashes |  |  |  |  |  |
| 1. Excessive sweating |  |  |  |  |  |
| 1. Trembling of hands |  |  |  |  |  |
| 1. Tingling feeling in fingers, arms, or legs |  |  |  |  |  |
| 1. Dry mouth |  |  |  |  |  |
| 1. Dizziness |  |  |  |  |  |
| 1. Confusion or feelings of unreality |  |  |  |  |  |
| 1. Nausea |  |  |  |  |  |
| 1. Headache |  |  |  |  |  |
| 1. Rustling sound in ears |  |  |  |  |  |
| 1. Fainting |  |  |  |  |  |
| 1. Lump in throat |  |  |  |  |  |
| 1. Having trouble swallowing |  |  |  |  |  |
| 1. Sore throat |  |  |  |  |  |

Persian version of psychosomatic symptoms questionnaire (PSQ-39)

| چقدر علائم زیر را در طول یک هفته گذشته (شامل روز جاری) تجربه کرده­اید. | | | | | |
| --- | --- | --- | --- | --- | --- |
| گزینه ها: | اصلا | کم | تا حدودی | زیاد | بسیار زیاد |
| 1. سر درد |  |  |  |  |  |
| 1. سرگیجه |  |  |  |  |  |
| 1. غش کردن یا بی­حال شدن |  |  |  |  |  |
| 1. تهوع |  |  |  |  |  |
| 1. وزوز گوش |  |  |  |  |  |
| 1. گیجی و منگی و داشتن احساسات غیر واقعی |  |  |  |  |  |
| 1. ناراحتی در معده |  |  |  |  |  |
| 1. شکم درد یا دل درد |  |  |  |  |  |
| 1. دل پیچه و گرفتگی روده و شکم |  |  |  |  |  |
| 1. نفخ یا ورم معده |  |  |  |  |  |
| 1. کم قوه بودن یا ضعف جسمانی |  |  |  |  |  |
| 1. احساس خستگی |  |  |  |  |  |
| 1. احساس بریدن و کم آوردن |  |  |  |  |  |
| 1. سرحال نبودن |  |  |  |  |  |
| 1. درد قفسه سینه |  |  |  |  |  |
| 1. سنگینی و فشار در قفسه سینه |  |  |  |  |  |
| 1. نامنظمی یا ضربان تند قلب |  |  |  |  |  |
| 1. تپش قلب |  |  |  |  |  |
| 1. سوزش سینه یا تیر کشیدن قلب |  |  |  |  |  |
| 1. احساس تنگی نفس یا نفس کم آوردن |  |  |  |  |  |
| 1. ناتوانی در تنفس عمیق یا نفس همراه با آه |  |  |  |  |  |
| 1. نفس نفس زدن |  |  |  |  |  |
| 1. درد عضلات |  |  |  |  |  |
| 1. درد استخوان و مفاصل |  |  |  |  |  |
| 1. کمر درد |  |  |  |  |  |
| 1. گردن درد |  |  |  |  |  |
| 1. احساس ضعف عضلانی |  |  |  |  |  |
| 1. سفتی و گرفتگی انگشتان، بازوها یا پاها |  |  |  |  |  |
| 1. لرزش دستان، بازوها یا پاها |  |  |  |  |  |
| 1. عرق کردن شدید |  |  |  |  |  |
| 1. لرز یا گر گرفتگی |  |  |  |  |  |
| 1. مور مور، کرخت یا بی حس شدن انگشتان، بازوها یا پاها |  |  |  |  |  |
| 1. خشکی دهان |  |  |  |  |  |
| 1. احساس وجود توده در گلو (گلوبوس) |  |  |  |  |  |
| 1. مشکل در بلع |  |  |  |  |  |
| 1. گلو درد |  |  |  |  |  |
| 1. فراموش­کار شدن |  |  |  |  |  |
| 1. تمرکز نداشتن |  |  |  |  |  |
| 1. افکار نامعلوم، مبهم و پریشان |  |  |  |  |  |

SF-36 questionnaire: <https://clinmedjournals.org/articles/jmdt/jmdt-2-023-figure-1.pdf>
